# Floral bud damage compensation by branching and biomass allocation in genotypes of *Brassica napus* with different architecture and branching potential

**DOI:** 10.3389/fpls.2015.00070

**Published:** 2015-02-24

**Authors:** Amélie Pinet, Amélie Mathieu, Alexandra Jullien

**Affiliations:** ^1^Institut National de la Recherche Agronomique, Unité Mixte de Recherche 1091, Environnement et Grandes CulturesF-78850 Thiverval-Grignon, France; ^2^AgroParisTech, Unité Mixte de Recherche 1091, Environnement et Grandes CulturesF-78850 Thiverval-Grignon, France

**Keywords:** winter oilseed rape, *Brassica napus*, architecture, biomass allocation, harvest index, allometry, plasticity, plant resilience

## Abstract

Plant branching is a key process in the yield elaboration of winter oilseed rape (WOSR). It is also involved in plant tolerance to flower damage because it allows the setting of new fertile inflorescences. Here we characterize the changes in the branching and distribution of the number of pods between primary and secondary inflorescences in response to floral bud clippings. Then we investigate the impacts of the modifications in branching on the biomass allocation and its consequence on the crop productivity (harvest index). These issues were addressed on plants with contrasted architecture and branching potential, using three genotypes (Exocet, Pollen, and Gamin) grown under two levels of nitrogen fertilization. Clipping treatments of increasing intensities were applied to either inflorescences or flower buds. We were able to show that restoration of the number of pods after clipping is the main lever for the compensation. Genotypes presented different behaviors in branching and biomass allocation as a function of clipping treatments. The number of fertile ramifications increased for the high intensities of clipping. In particular, the growth of secondary ramifications carried by branches developed before clipping has been observed. The proportions of yield and of number of pods carried by these secondary axes increased and became almost equivalent to the proportion carried by primary inflorescences. In terms of biomass allocation, variations have also been evidenced in the relationship between pod dry mass on a given axis and the number of pods set, while the shoot/root ratio was not modified. The harvest index presented different responses: it decreased after flower buds clipping, while it was maintained after the clipping of the whole inflorescences. The results are discussed relative to their implications regarding the identification of interesting traits to be target in breeding programs in order to improve WOSR tolerance.

## Introduction

**Figure 1:**
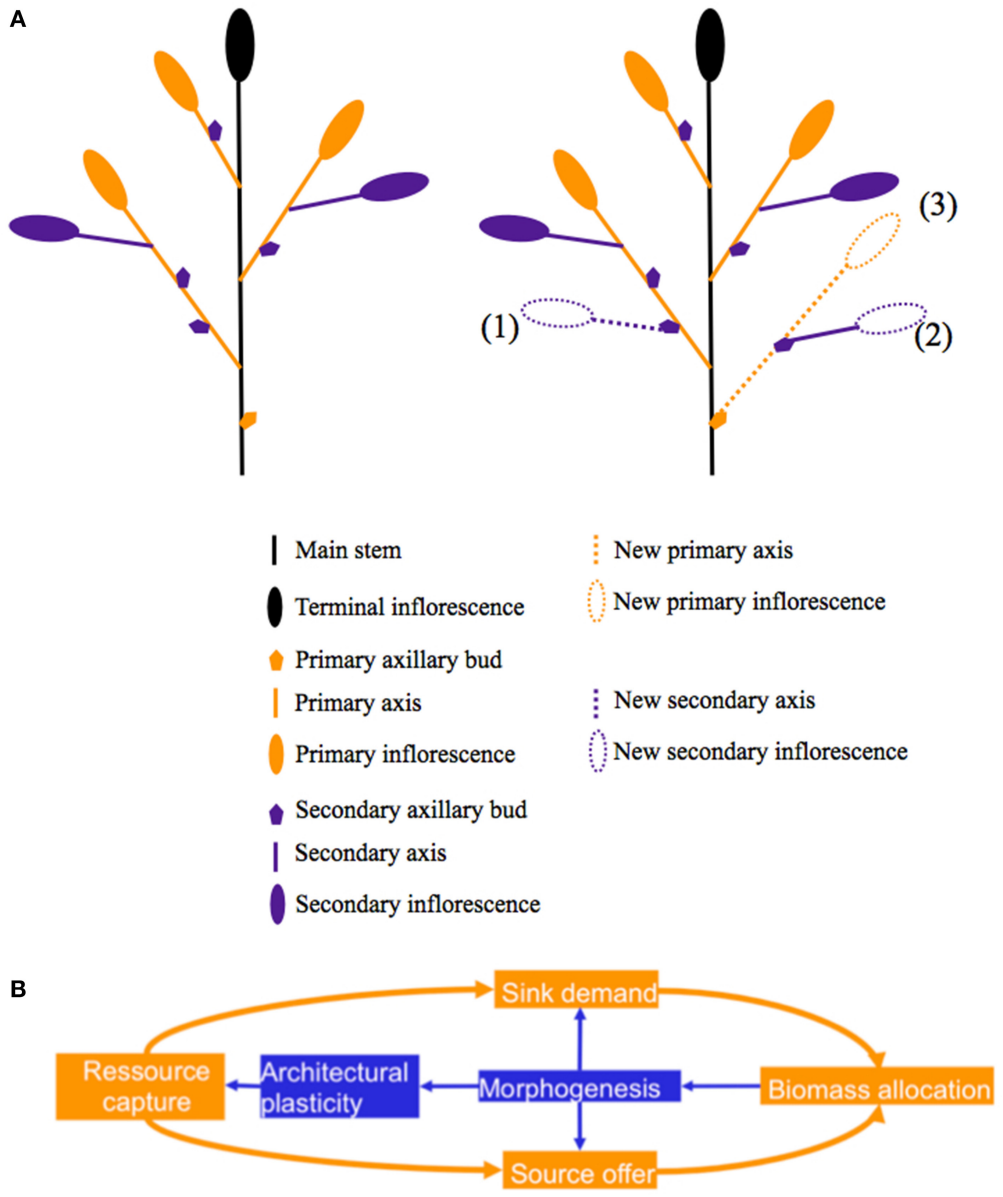
**(A) Topology of the aerial parts of a WOSR plant**. Left: topology before floral bud damage, right: after floral bud damage newly formed pods may be located on either existing inflorescences or on new inflorescences. New inflorescences may be carried by existing primary axes (1) or by new ones (2,3). **(B)** Diagram of the interaction between branching pattern and biomass allocation.

Branching is an important component of the yield of winter oilseed rape (WOSR, McGregor, 1981; Leterme, 1985; Lu et al., 2011). The final architecture of the inflorescences and subsequently of the yield depends on the ontogenetic dynamics of the apical and axillary meristems giving rise to branches of increasing order, as has been described for different crops and wild species by Moulia et al. (1999a,b), Van Minnebruggen et al. (2014), and Park et al. (2014). In the specific case of WOSR, floral initiation on the apical meristem occurs during the winter between the mid-November and mid-January (Tittonel, 1990). At the end of winter, during plant bolting and stem elongation, and depending on the environment (and notably plant density—Retuerto and Woodward, 2001) varying numbers of buds outgrow to produce fertile branches. Flowering starts on the main inflorescence and propagates basipetally from the youngest to the oldest branches of the plant (Jullien et al., 2011). Generally final plant yield is distributed between the apical inflorescence (approximately 10–20%—Leterme, 1985; Allirand et al., 2011) and lateral branches each bearing a terminal inflorescence (primary inflorescence) as well as varying numbers of secondary branches with their own terminal inflorescence (secondary inflorescences—Figure 1A).

Branching is highly plastic as a function of genotype and environment (Pinet, 2010; Lei et al., 2014; Van Minnebruggen et al., 2014). The outgrowth of buds responds to different environmental signals such as light quantity, light quality and nitrogen (Evers et al., 2011; Furet et al., 2014; Lei et al., 2014; Park et al., 2014). It is also governed by a complex hormonal regulation (Janssen et al., 2014) and requires the allocation of carbohydrates (Mason et al., 2014). Thanks to its plasticity, branching is often involved in the response of plants to environmental constraints, and notably to flower damage (Sadras, 1996; Tiffin, 2000). In WOSR, floral bud damage may be due to pests (Lerin, 1987; Brandt and Lamb, 1994; Nilsson, 1994) or environmental constraints (Morrison, 1993; Lardon and Triboi-Blondel, 1995; Annisa et al., 2013) and can cause potentially dramatic yield losses. In particular, before flowering, WOSR plants may undergo massive floral bud losses due to pollen stealing by pollen beetles (*Meligethes aeneus* L—Lerin, 1987). Pollen beetles cause substantial and sometimes devastating damage (yield reduction as high as 70% has been recorded—Nilsson, 1994).

Compensation is often achieved by the production of new floral buds more than by the increase of seed weight (Williams and Free, 1979; Lerin, 1987; Tommey and Evans, 1992; Nilsson, 1994). Compensating floral buds may be produced on inflorescences carried by either existing or new branches (Tatchell, 1983; Nilsson, 1994). The setting of new branches and inflorescences can compensate for floral bud losses but may also modify final biomass partitioning within the plant. In particular, it may impact the ratio between seed weight and total plant weight, the so-called Harvest Index (HI), which is an important criterion of crop productivity. The interaction between the plasticity of the branching and the biomass re-allocation is thus a component of plant tolerance to flower damage (Tiffin, 2000). We have assumed that these interactions between architecture and morphogenesis may vary according to genotype and environment. This variability can be used to identify traits, which could be targeted in breeding programs in order to improve WOSR tolerance to flower damage.

The interaction between branching and biomass allocation was studied through the pruning of inflorescences and branches by Bennett et al. (2012) for *Brassica napus, Brassica rapa*, and *Arabidopsis thaliana*. Their results showed that morphogenesis controls the allocation of biomass between vegetative and reproductive organs (Figure 1B). While their results focused on the impact of the removing of lateral branches on the architecture of the apical inflorescence (elongation, size of pods, seed weight), we proposed to extend this study and to evaluate (i) the modification of both the global pattern of ramification and the yield distribution between branches and (ii) the implications in terms of biomass allocation in response to different intensities of inflorescence and floral bud pruning, with particular focus on WOSR. Our hypotheses were (i) that an increased proportion of yield borne by secondary axes may result in compensation, (ii) that at the scale of the axis, biomass allocation rules are conserved, and particularly the allometric relationships between the number of pods and the pod yield, and (iii) that at the plant scale an increase in biomass investment into structural tissues (new branches) and a reduction in plant HI will be observed.

These issues were addressed on plants with contrasted architecture and branching potential, using three genotypes (Exocet, Pollen, and Gamin) grown under two levels of nitrogen fertilization. With regards to the use of a low nitrogen fertilization treatment, we hypothesized that the plant ability to grow new branches and thus to compensate the floral damage will be lower. Clipping treatments of increasing intensities were applied to either inflorescences or flower buds.

## Materials and methods

### Experimental design

The experiments were carried out in 2007–2008 (Y1–ClipInflo experiment) and 2008–2009 (Y2—ClipFB experiment) in the Paris region of France (48.9°N, 1.9°E). The plants were grown under field conditions. Seeds were sown on 4th Sept. 2007 and 7th Sept. 2008 at a density of 50 seeds.m^−2^. We designed different combinations of variety and nitrogen fertilization (VN combinations) in order to generate a broad range of dynamics of reproductive morphogenesis. Three varieties with contrasting architectures were studied: Pollen, Exocet and Gamin. Pollen and Exocet usually bear fewer axes than Gamin (6–9 axes vs. 10–15 axes). Pollen and Exocet present a standard height at harvest (1.80 m) while Gamin is a half-dwarf variety (1.30 m). In addition, Pollen starts flowering earlier than Gamin and Exocet. Furthermore, Exocet is a hybrid while Pollen and Gamin are inbred lines. Finally, Gamin produces more floral buds and pods than Exocet and Pollen (approximately 1000 floral buds per plant for Gamin compared to 600 floral buds per plant for Pollen—Pinet, 2010). Two levels of nitrogen fertilization were used as treatments: high (HN—100 kg.ha^−1^ on 1st Mar. 2008 for Y1 and 1st Mar. 2009 for Y2 and 40 kg.ha^−1^ on 17th Mar. 2008 for Y1 and 50 kg.ha^−1^ on17th Mar. 2009 for Y2) and low (LN—70 kg.ha^−1^ on 17th Mar. 2008 for Y1 and 40 kg.ha^−1^ on 17th Mar. 2009 for Y2). In Y1 (ClipInflo experiment), four VN were used: Exocet, Pollen and Gamin with high nitrogen fertilization (EHN, PHN, GHN, respectively) and Exocet with low nitrogen fertilization (ELN). In Y2 (ClipFB experiment), six VN were used: Exocet, Pollen and Gamin with both high (EHN, GHN, PHN, respectively) and low nitrogen fertilization (ELN, GLN, PLN, respectively). Soil characteristics (water and nitrogen contents) were assessed for their similarity (data not shown). Thus, the individual plant is the replicate.

### Clipping treatments

Clipping was performed on individual inflorescences and data were recorded at the plant scale. The plants were subjected to either inflorescence (ClipInflo) or to floral bud (ClipFB) clipping. In ClipInflo, three intensities of inflorescence clippings were applied: no clipping (Control), clipping of the terminal inflorescence (ClipI0) and clipping of the terminal inflorescence and the four most apical inflorescences (ClipI4). Inflorescences were cut before flowering (level 55 on the BBCH scale—Zadoks, 1974) at the axil of the leaf from which they originated. ClipInflo was applied to four VN combinations: EHN, ELN, GHN, and PHN. The ClipI0 treatment represented from 5% (for EHN) to 10% (for ELN and PHN) of the total number of inflorescences in the different VN combinations. For ClipI4 treatment, the percentage of clipped inflorescences represented from 33% (GHN) to 50% (EHN) of the total number of inflorescences in the different VN combinations.

In ClipFB, three intensities of floral bud clippings were carried out as follows: no clipping (Control), clipping of 50 floral buds on the main inflorescence and 20 floral buds on each of the four most apical inflorescences (ClipFB4), and clipping of all floral buds on the seven most apical inflorescences (ClipFB7). Clipping was applied before flowering (level 55 on the BBCH scale, on 17th Apr. 2009) to floral buds with a minimum diameter of 3 × 10^−3^m in order to avoid damage to the meristems. ClipFB was carried out on 6 VN combinations: EHN, ELN, GHN, GLN, PHN, and PLN. For ClipFB4, the percentage of clipped floral buds varied according to the VN combinations (from 23% for GHN to 60.5% for GLN). With ClipFB7, the percentage of clipped floral buds ranged from 67% (GHN) to 94% (PLN).

### Measurements

In ClipInflo, the number of plants per treatment was five. At harvest (23rd May 2008), the number of fertile primary and secondary inflorescences per plant was counted, as was the total number of pods per primary axis (pooling the pods borne by primary and secondary inflorescences on a given primary axis). Each plant was divided into six compartments for dry weight measurements: the roots, the main stem, the vegetative parts (stem and leaves) of primary and secondary axes, the reproductive parts (pods) of primary and secondary axes. Each part was oven-dried (48 h at 80 °C) and then weighed.

In ClipFB, for all VN and treatments, the number of pods and pod weight per plant as well as the number of primary axes and the dry matter by compartment were counted and measured on a sample of 10 or 13 plants. The number of pods and the pod weight per axis were measured on a sub-sample of 3 plants. In addition, on another subsample of three plants, and only for the Control and ClipFB7 treatments of GHN, GLN, PHN and PLN, the number of pods and the pod weight per axis as well as the number of secondary axes were measured and counted. Details of experiments are summarized in Table 1.

**Table 1:** **Treatments and measurements carried out during the 2 years of the experiment**.

				**Size of the main sample**			**Size of the sub-sample**	
**Years**	**Experiments**	**VN combinations**	**Clipping treatments**	**Number of pods and pod weight per plant**	**Number of primary axes**	**Dry matter by compartment**	**Number of pods and pod weight per axis**	**Number of secondary axes**
Y1 2007–2008	ClipInflo	EHN, ELN, GHN, PHN	Control	5	5	5	5	5
			ClipI0	5	5	5	5	5
			ClipI4	5	5	5	5	5
Y2 2008–2009	ClipFB	EHN, ELN	Control	10	10	10	3	0
			ClipFB4	10	10	10	3	0
			ClipFB7	10	10	10	3	0
		GHN, GLN	Control	13	13	13	6	3
			ClipFB4	10	10	10	3	0
			ClipFB7	13	13	13	6	3
		PHN, PLN	Control	13	13	13	6	3
			ClipFB4	10	10	10	3	0
			ClipFB7	13	13	13	6	3

*VN combinations correspond to the Variety and Nitrogen combinations. E, G, and P mean Exocet, Gamin and Pollen varieties, respectively. HN and LN correspond to high and low nitrogen fertilizations. EHN, Exocet High Nitrogen; ELN, Exocet Low Nitrogen; GHN, Gamin High Nitrogen; GLN, Gamin Low Nitrogen; PHN, Pollen High Nitrogen; PLN, Pollen Low Nitrogen. Inflorescence clipping experiment (ClipInflo) corresponds to the clipping of the apical inflorescence (ClipI0) and to the clippings of the apical and the four most apical inflorescences (ClipI4). Bud clipping inflorescences (ClipFB) corresponds to the clippings of buds on each of the four most apical inflorescences (ClipFB4) and on the seven most apical inflorescences (ClipBF7). Compartments are the roots, the main stem, the vegetative parts (stem and leaves) and the reproductive parts (pods) of primary and secondary axes*.

### Data processing

#### Tolerance indices

Tolerance indices were calculated with respect to grain yield, number of pods and weight of a thousand pods. The index was calculated as the ratio between the value for an individual plant and the mean value of the control treatment of the corresponding VN combination (Agrawal et al., 1999; Wise et al., 2008).

#### Distribution of pods throughout plant architecture

In order to assess the distribution of pods throughout plant architecture, we defined the following four classes: pods carried by existing axes on either primary (Class1) or secondary inflorescences (Class2), and pods carried by new axes on either primary (Class 3) or secondary inflorescences (Class 4) (Figure 1A). For each experiment and VN combination, the mean number of fertile primary axes for the Control treatment was calculated. Then, for each clipping treatments and each plant, the numbers of existing and new fertile primary axes were determined in comparison to the Control treatment: new fertile axes correspond to axes that are present in clipped plants and absent in the intact ones.

#### Biomass allocation

At the axis scale, biomass allocation between the number of pods and pod dry mass was assessed by fitting a mixed linear model between the logarithms of the two variables. The “Plant” factor forms the random part; while the “Clipping” and “Pod dry mass” factors were considered as fixed factors of the mixed model (Bolker et al., 2009). Assessment of the statistical significance of the “Clipping” factor was made using *F*-tests (*P* < 0.05). At the plant scale, the percentage in dry matter of each of the six compartments was calculated to determine the relative allocation between vegetative and reproductive organs on one hand and between first order and second order branches on the other hand. The ratio between shoot dry matter and root dry matter was also calculated. The harvest index was calculated ultimately from the ratio between the pod dry matter and the total aerial dry matter.

#### Statistical tests

***Classification trees***. In the first part of this paper, we used a tree-structured recursive partitioning method to describe the conditional distribution of the tolerance in grain yield given the status of two covariates that are the tolerances in terms of number of pods and of pod weight. A detailed explanation of the conditional inference tree method is given by Strobl et al. (2008) and Hothorn et al. (2006). Roughly, the algorithm works as follows: firstly, the global null hypothesis of independence between any of the input variables (here, the tolerance indices in number of pods and pod weight) and the response (the tolerance index in grain yield) is tested. The stop criterion is based on multiplicity adjusted *p*-values (“Bonferroni”). The criterion is maximized, i.e., 1—*p*-value is used. A split is implemented when the criterion exceeds a threshold. For example, when the threshold is equal to 0.95, the *p*-value must be smaller than 0.05 to split this node. Secondly, a binary split in the selected input variable is implemented. These two steps are repeated until the null hypothesis cannot be rejected, i.e., the criterion does not exceed the threshold. This statistical approach ensures that the right sized tree is grown and that no pruning or cross-validation or whatsoever is needed.

***Mann–Whitney tests***. Due to the small sample sizes, the branching and biomass allocation variables did not meet the assumptions of normality so that their responsiveness to the clipping treatments was examined using Mann–Whitney tests (Sokal and Rohlf, 1995).

***Software***. Statistical treatments were performed using the statistical program R.

For the sake of legibility and to lighten the manuscript, not all the VN are presented in the figures. When the results concerns both experiments, only the common VN are presented (EHN, ELN, GHN, and PHN). The other VN combinations are shown in the SI. However, in the specific cases when the two experiments or the VN combinations show difference in the magnitude of the responses, the results of all the VN combinations are presented.

## Results

### What were the respective contributions of the number of pods and of the pod weight to the plant compensation?

**Figure 2:**
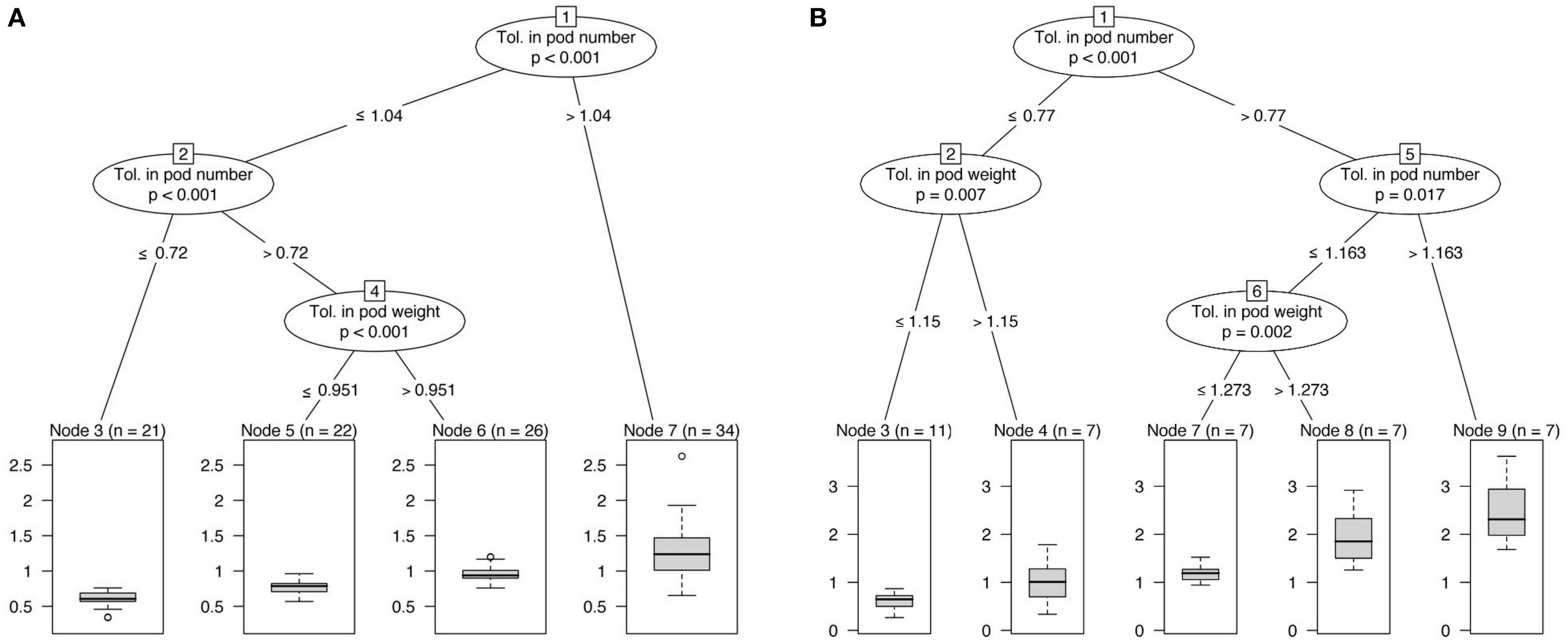
**Classification trees based on tolerance indices of seed yield, number of pods and pod weight. (A)** ClipFB, **(B)** ClipInflo.

In the two experiments, the tolerance in terms of number of pods is the covariate showing the strongest association with the tolerance in grain yield. This result highlighted the key role of the production of new pods in WOSR compensation in response to flower damage (Figure 2). When considering both experiments, compensation in seed yield was observed in most of the plants (71% for ClipInflo, 73% for ClipFB). Tolerance indices in the number of pods of 77% (ClipInflo) and 104% (ClipFB) were enough to compensate with respect to seed yield. Below these thresholds, different patterns were observed according to clipping treatments and that corresponds to different combinations of tolerance indices for the number of pods and the pod weight. For ClipFB, a minimal tolerance index of 72% in the number of pods is necessary, below this value no compensation in the seed yield was observed. Between 72 and 104%, a tolerance index of 95% with respect to the pod weight was necessary to compensate in seed yield. For ClipInflo, for a tolerance index below 77% for the number of pods, a tolerance index of at least 115% (overcompensation) with respect to the pod weight was necessary to compensate in seed yield. No threshold in the tolerance index of the number of pods was detected below which there was no possible compensation in seed yield (whatever the compensation in the pod weight). It is although noteworthy that 53% of the plants under ClipInflo overcompensated i.e., had a tolerance index higher than one.

No significant difference in the tolerance in seed yield was observed according neither to genotype nor to nitrogen. Only ELN had a noteworthy low tolerance in seed yield (0.69 on average over ClipFB4, ClipFB7, ClipI0, and ClipI4) but the difference with the Control was not significant due to an important range of variation.

### Was there any modification in plant branching in response to the clipping treatments?

**Figure 3:**
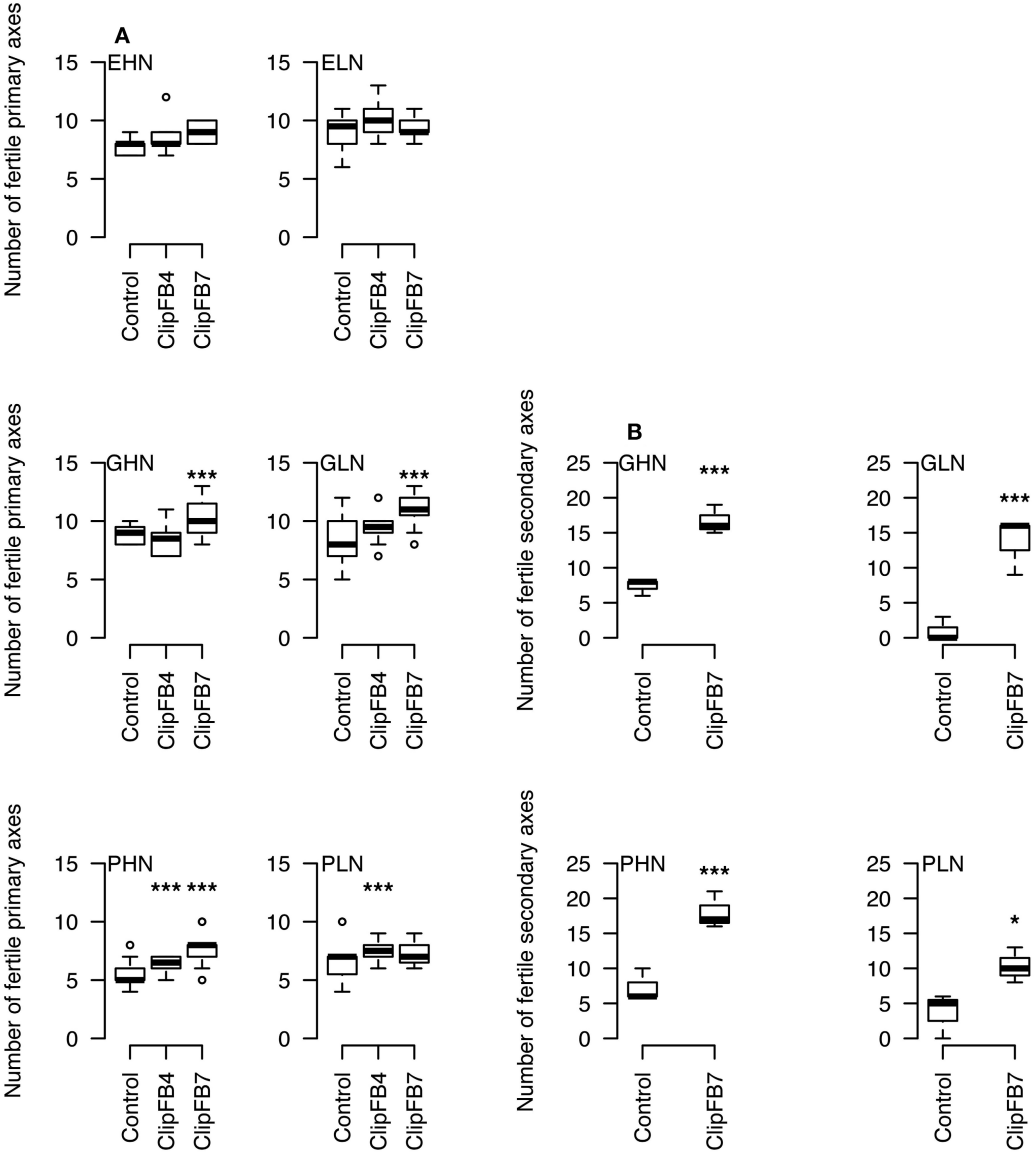
**Number of fertile primary (A) and secondary (B) axes per plant in ClipFB experiment for EHN, ELN, GHN, GLN, PHN and PLN**. ^***^0.05 / ^*^ 0.15 using Mann–Whitney tests. The meanings of the acronyms of the VN combinations are detailed in Table 1.

A global upward trend in the number of fertile primary axes has been observed in response to floral bud clipping of increasing intensities (ClipFB, Figure 3A). This increase was significant for plants under ClipFB4 PHN (+1.5, *P* < 0.05), and PLN (+1, *P* < 0.05) as well as for ClipFB7 GHN (+1, *P* < 0.05), GLN (+3, *P* < 0.05), and PHN (+3, *P* < 0.05). In parallel, the number of fertile secondary axes per plant increased significantly for plants of GHN, GLN, PHN, and PLN in response to ClipBF7 (+8, +16, +11 with *P* < 0.1 and +5 inflorescences with *P* < 0.15, respectively—Figure 3B).

**Figure 4:**
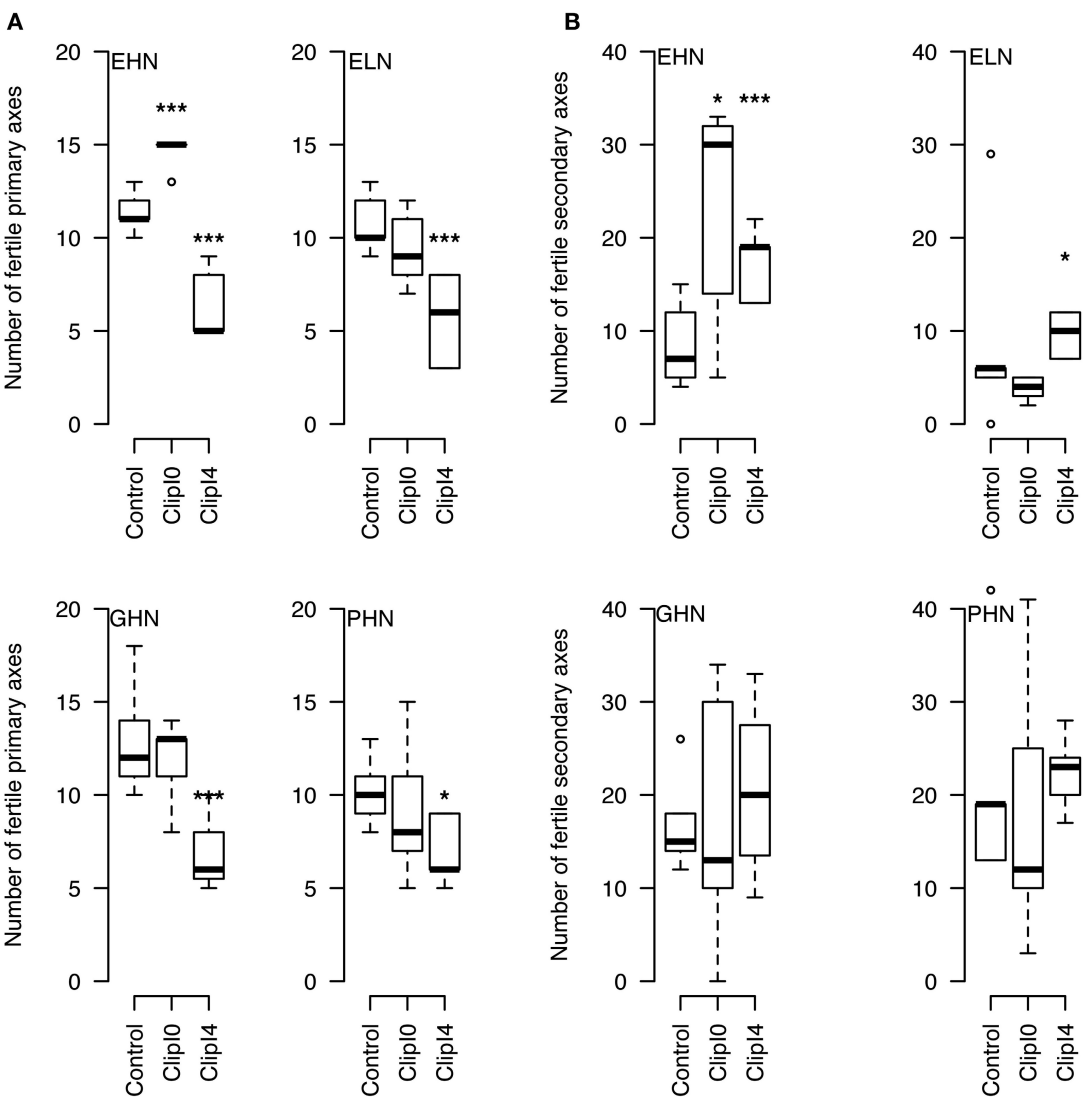
**Number of fertile primary (A) and secondary (B) axes per plant in ClipInflo experiment for EHN, ELN, GHN and PHN**. ^***^ 0.05 / ^*^ 0.15 using Mann–Whitney tests. The meanings of the acronyms of the VN combinations are detailed in Table 1.

The clipping of the apical inflorescence (ClipI0) did not modify significantly the number of fertile primary axes (except for EHN where 4 new basal fertile axes were observed, *P* < 0.05,—Figure 4A). The intensive clipping (ClipI4) reduced by 4 the final number of fertile primary axes, i.e., no new fertile primary axis was produced to compensate the initial loss (Figure 4A). The interquartile range of the number of fertile secondary axes was dramatically more variable compared to primary axes (except for ELN for which the number of secondary axes was very low). Increase in the number of fertile secondary axes was significant for EHN (+23 inflorescences in Clip I0 and +12 in inflorescences ClipI4) and ELN (+4 inflorescences—Figure 4B).

### How far do these new axes modify plant yield architecture?

During both experiments, we observed a shift in pod yield distribution from the primary to the secondary inflorescences. For ClipFB, secondary inflorescences carried 2.6% of yield in control plants. This proportion increased significantly in response to ClipFB4 (17%, *P* < 0.05) and to ClipFB7 (43%, *P* < 0.05). For plants of ClipInflo, the same observation was made with 26% of yield carried by secondary inflorescences in the control plants, compared to 25.4% (pv = ns) and 42.5% (*P* < 0.05) in ClipI0 and ClipI4 treatments.

**Figure 5:**
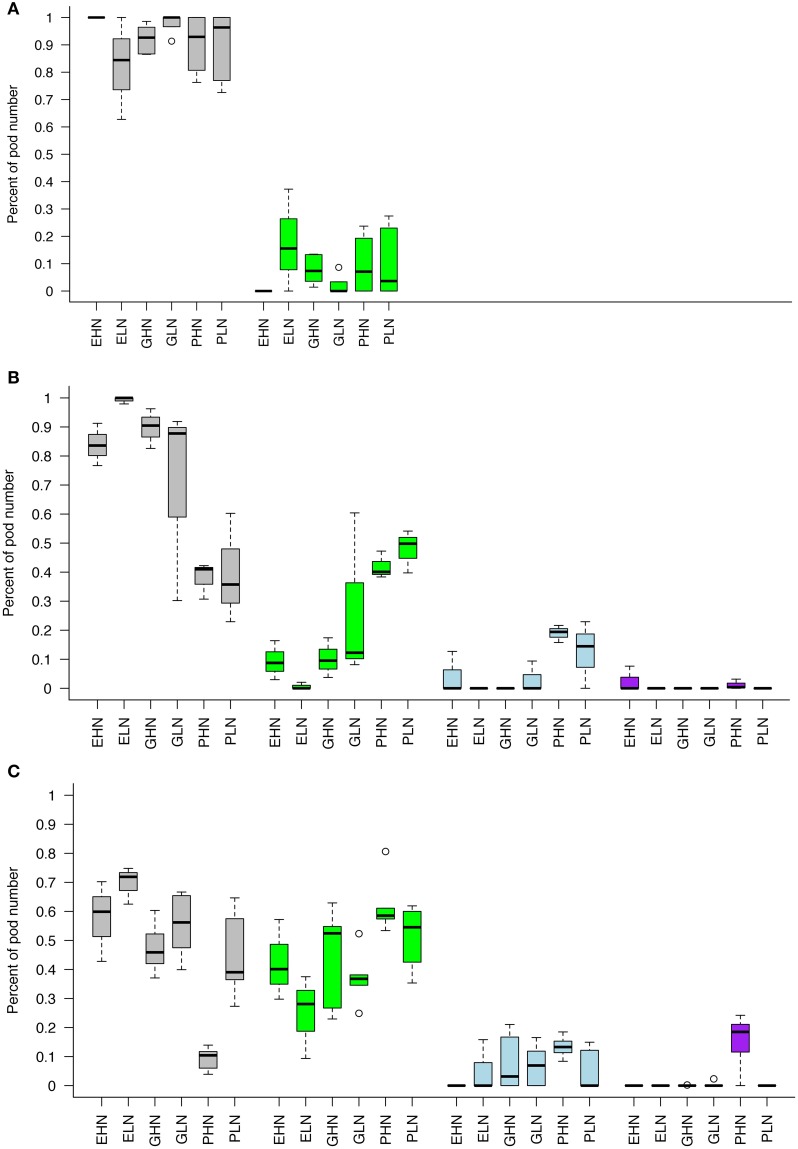
**Pods distribution between different axes of the plant for ClipFB**. Control **(A)**, ClipFB4 **(B)**, and ClipFB7 **(C)**. Gray boxplots correspond to pods of primary inflorescences carried by existing primary axes. Green boxplots correspond to pods of secondary inflorescences carried by existing primary axes. Blue boxplots correspond to pods of primary inflorescences carried by new primary axes. Purple boxplots correspond to pods of secondary inflorescences carried by new primary axes. Results are expressed as a percentage of the total number of pods. The meanings of the acronyms of the VN combinations are detailed in Table 1.

For the sake of clarity, only the data concerning the topology of distribution of pods in ClipFB are presented here in Figure 5 (see Figure 1 in SI for ClipInflo). The distribution of pods was modified for some VN of ClipFB4: EHN, GLN, PHN and PLN had a reduced proportion of pods on the primary inflorescences of respectively, −17, −12, −56, and −60%. The ranges of variation of the box plot were also increased compared to Control. These decreases were compensated by both an increase in the proportion of pods carried by the secondary inflorescences and the appearance of some new axes. The appearance of new axes was observed for EHN, GLN, PHN, and PLN even if this was more important for PHN (+2%) and PLN (+14.5%).

The modifications observed in the plants under ClipFB4 were amplified in the plants under ClipFB7: the decrease in the proportion of pods carried by the primary inflorescence was all the most important and concerned all the VN: EHN (−41%), ELN (−29%,) GHN (−51%), GLN (−43%), PHN (−87%), PLN (−56%) compared to Control. In the same manner the proportion of pods carried by the secondary inflorescences increased for all VN (+40, +23, +48, +36, +54, +49% for ELN, GHN, GLN, PHN, and PLN respectively), and the appearance of new primary and secondary axes were observed for GHN, GLN and PHN (+3, +7, +13.3% for GHN, GLN, PHN on new primary inflorescences and +18.5% for PHN on new secondary inflorescences). The differences in the response of the different VN to ClipFB are coherent with the results obtained on the number of axes (Figure 3): plants of Gamin and Pollen were more reactive to clipping than plants of Exocet in response to ClipFB.

In the Control of ClipInflo (Figure 1 in SI), the primary inflorescences produced the most important share of pods on the plant (from 66.5% for GHN to 94.5% for ELN), except for PHN (54%). It is also noteworthy that the production of pods on secondary inflorescences was much higher than in ClipFB experiment in three out of four VN (EHN, GHN, PHN).

In response to ClipI0, the proportion of pods carried by the primary inflorescence decreased only for plants under EHN (−45%). For the three other VN combinations, the production of pods on primary and secondary inflorescences on existing axes remained constant but with a higher range of variability. Pods carried by primary and secondary inflorescences on new axes (Class 3 and Class 4, respectively) were of less importance, except for EHN with 15% and 11% of the total number of pods carried by primary and secondary inflorescences, respectively.

In response to ClipI4, part of pods produced by primary inflorescences on existing axes decreased (−39% for EHN, −18% for ELN, −29% for GHN, −10% for PHN compared to Control). Pods carried by secondary inflorescences on existing primary axes accounted for a higher share for the four VN combinations (+25.5, +16.5, +22, +8.5% for EHN, ELN, GHN, and PHN, respectively). There was no pod carried by primary and secondary inflorescences on new axes.

Concerning the differences between genotypes behaviors under ClipInflo, Exocet was more reactive than Gamin and Pollen. This is coherent with results obtained on the number of axes (Figure 4) but different from results obtained for plants under ClipFB.

### How is biomass allocation modified in response to these changes in pod distribution and yield architecture?

#### Allocation at the branch scale

**Figure 6:**
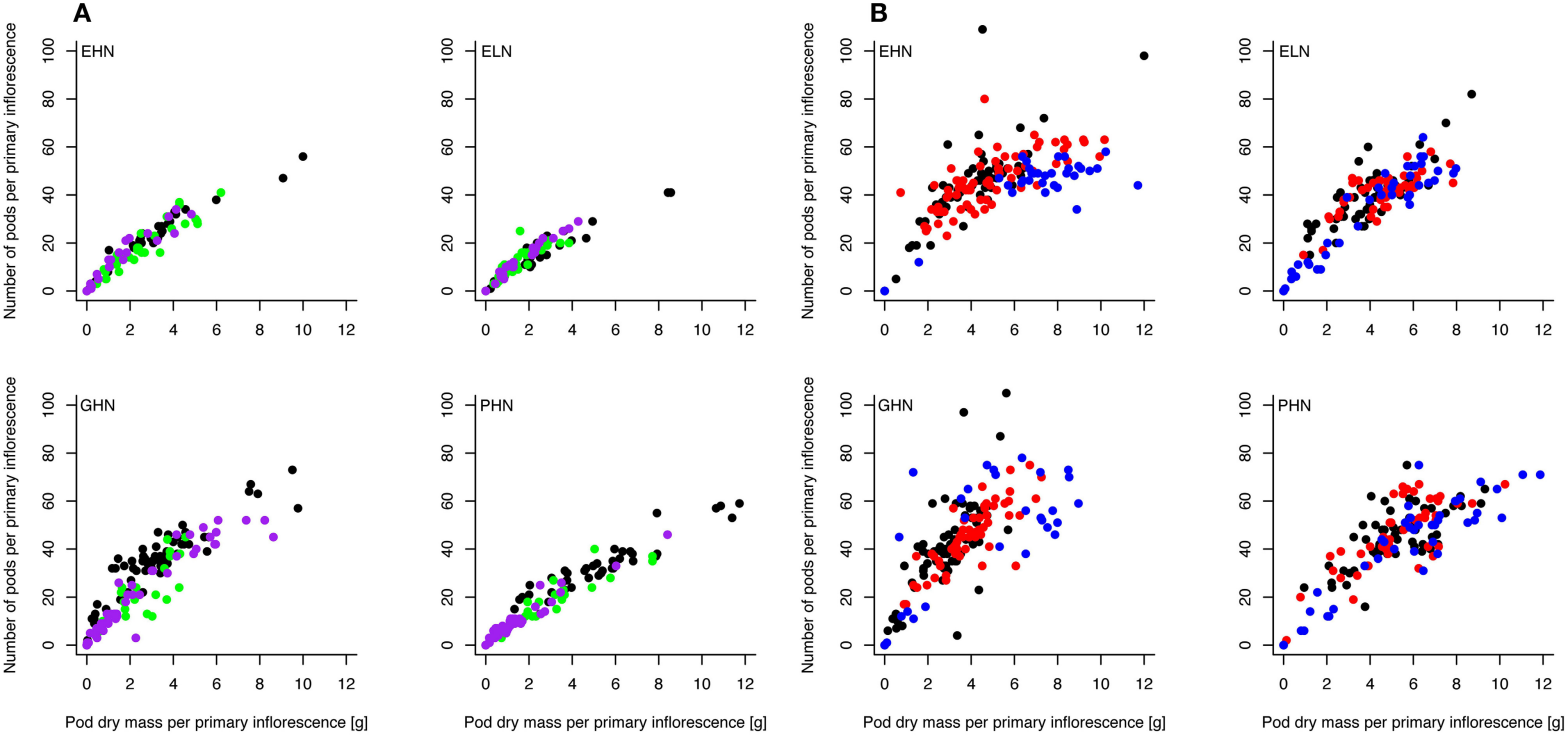
**Relationship between the number of pods and the total pod dry mass on the primary inflorescences for EHN, ELN, GHN, and PHN of ClipFB (A) and ClipInflo (B)**. Black, green and purple dots correspond to Control, ClipFB4 and ClipFB7 treatments, respectively. Black, red and blue dots correspond to Control, ClipI0 and ClipI4 treatments, respectively. The meanings of the acronyms of the VN combinations are detailed in Table 1. Linear mixed-models were used to assess the significance of the results.

The relationship between pod dry mass and number of pods, respectively, was analyzed separately for ClipFB and ClipInflo and for the primary (Figure 6 and Figure 2 in SI) and secondary (Figure 3 in SI) inflorescences. A slow-down in the increase in the number of pods above a certain pod dry mass was observed for the primary inflorescences of ClipInflo for some VN combinations and clipping intensities (Figure 6B). Above this threshold, biomass will be allocated to existing pods to increase their weight instead than to the meristem to produce new pods what is a shift in the biomass allocation. The number of pods increased linearly with pod dry mass in secondary inflorescences indicating no slow-down in the number of pods for ClipFB and ClipInflo (Figure 3 in SI). The mixed linear model fitted to data (after logarithmic transformation) showed significant variation in the slopes and the intercepts for 17 of the 40 combinations of VN and clipping treatments of ClipFB and ClipInflo. The main effect of the clipping treatments was a shift in the x-axis that concerned an increase in pod dry mass, which was between 60 and 100%. In comparison, significant variations detected in the slopes ranged from 26 to 53%, which is much smaller.

#### Shoot/root

Under ClipInflo, similar variations in the shoot/root ratio were found with all the treatments, i.e., a global upward trend with increasing clippings but that was not significant. This result is illustrated for the two experiments for EHN, ELN, GHN, and PHN in Table 2. Under ClipFB, the results were more variable and no significant variation was identified. The shoot/root ratio tended to increase in line with clipping intensity under ClipInflo for the VN of high nitrogen treatment (EHN, GHN and PHN). The shoot/root was higher under high nitrogen treatment (EHN) than under low nitrogen treatment (ELN) in ClipInflo and ClipFB, which is a classic response by the shoot/root ratio to N starvation.

**Table 2:** **Shoot:root ratio (g.g^−1^) for the EHN, ELN, GHN and PHN combinations of ClipInflo and ClipFB experiments**.

**VN**	**Control**	**ClipFB4**	**ClipFB7**
EHN	12.74 [2.15–19.52]	13.39 [2.61–21.23]	17.17 [2.56–22.02]
ELN	10.56 [2.65–14.5]	8.84 [2.86–13.46]	9.97 [2.85–14.34]
GHN	11.95 [2.15–17.58]	10.25 [2.3–16.02]	10.98 [2.5–18.94]
GLN	9.5 [2.011–13.11]	8.32 [2.4–12.38]	8.71 [2.72–13.53]
PHN	12.38 [2.38–17.54]	11.3 [2.47–18.34]	13.86 [2.6–17.86]
PLN	7.8 [2.32–10.59]	8.65 [2.85–13.28]	8.39 [2.9–12.16]
**VN**	**Control**	**ClipI0**	**ClipI4**
EHN	15.86 [13.79–19.63]	16.66 [12.0–20.18]	17.19 [13.76–19.90]
ELN	9.31 [7.80–12.72]	10.97 [9.03–12.91]	10.13 [7.13–13.34]
GHN	11.33 [11.14–16.5]	12.33 [10.7–14.87]	10.94 [8.47–12.0]
PHN	14.83 [10.75–17]	13.1 [10.9–17.24]	15.47 [11.85–17.8]

*Data in the table indicate the median value with minimum and maximum in brackets. Mann–Whitney tests were used to assess the significance of the results*.

#### Harvest index

The harvest index values measured for the two clipping treatments are presented for all the VN combinations (Table 3). Results indicate a difference between the two clipping treatments in terms of crop competitivity. For ClipFB, the harvest index decreased significantly for all the VN combinations (−9% for ClipFB4 and –13% for ClipFB7). This decrease in the harvest index resulted from the significant increase in the aerial vegetative biomass (except for ClipFB4 and ClipFB7 ELN and ClipFB4 GHN), while pod dry mass was not affected (ClipFB4 GLN; ClipFB7 GHN, GLN, PHN), decreased (ClipFB4 EHN, ELN, GHN; ClipFB7 EHN, ELN, PLN) or increased (ClipFB4 PHN, PLN). The increase in vegetative biomass could be related to the production of new secondary axes.

**Table 3:** **Harvest index for all the VN combinations of ClipInflo (EHN, ELN, GHN and PHN) and ClipFB (EHN, ELN, GHN, GLN, PHN, PLN) experiments**.

**VN**	**Control**	**ClipFB4**	**ClipFB7**
EHN	0.64 [0.61–0.69]	0.58 ^***^ [0.51–0.63]	0.56 ^***^ [0.48–0.59]
ELN	0.62 [0.54–0.64]	0.57 ^***^ [0.46–0.59]	0.56 ^***^ [0.52–0.69]
GHN	0.68 [0.57–0.70]	0.65 ^***^ [0.55–0.68]	0.60 ^***^ [0.51–0.62]
GLN	0.65 [0.62–0.69]	0.60 ^***^ [0.52–0.62]	0.56 ^***^ [0.52–0.61]
PHN	0.67 [0.55–0.72]	0.62 ^***^ [0.59–0.64]	0.58 ^***^ [0.50–0.60]
PLN	0.64 [0.59–0.7]	0.55 ^***^ [0.52–0.59]	0.58 ^***^ [0.45–0.59]
**VN**	**Control**	**ClipI0**	**ClipI4**
EHN	0.56 [0.55–0.58]	0.56 [0.51–0.59]	0.56 [0.51–0.67]
ELN	0.58 [0.49–0.73]	0.54 [0.51–0.57]	0.52 [0.50–0.56]
GHN	0.55 [0.49–0.63]	0.65 ^***^ [0.60–0.67]	0.64 [0.57–0.66]
PHN	0.53 [0.53–0.58]	0.54 [0.50–0.57]	0.55 [0.53–0.57]

Data in the table indicate the median value with minimum and maximum in brackets.

*** *indicates significant difference at P<0.05 using Mann–Whitney tests*.

For ClipInflo, the harvest index was not modified by the clipping treatments, except for ClipI0 GHN for which it increased. Aerial vegetative biomass was not modified by the clipping treatment for the four VN (data not shown). Consequently, the variations in the harvest index were closely related to variations in pod dry mass. This was specifically the case for GHN for which the increase in the harvest index was related to a marked increase in pod dry mass (tolerance in pod yield is equal to 1.32).

## Discussion

We have shown that flower damage modifies yield architecture and its components. In particular we were able to show that restoration of the number of pods is the main lever for the compensation in yield. New pods were mainly set on primary inflorescences or on new secondary inflorescences carried by existing primary branches. Newly produced primary branches with fertile pods were observed for the highest clipping intensities. The different clipping treatments reduced the predominance of the primary inflorescences compared to secondary inflorescences (from 90% for the Control to 50% for the most severe cutting treatment). In the specific case of inflorescence clipping, a high share of overcompensation was observed what is a classic response by the rapeseed to the suppression of apical dominance.

These results were consistent with those obtained by Williams and Free (1979), Lerin (1987) and Nilsson (1994) who had already evidenced the importance of the number of pods for the compensation. Tommey and Evans (1992) also demonstrated that floral bud clippings on primary axes increased the yield carried by the remaining intact primary axes. However, they did not describe the spatial distribution of these new pods.

Two factors, the variety an the nitrogen fertilization, were defined to generate a wide range of branching potential. With regards to nitrogen, we were expecting different responses in branching and compensation for the high and low nitrogen fertilization because nitrogen deficiency is known to reduce the branching. Indeed it can be assumed that the ability of a plant to produce new inflorescences is related to both its architecture and nutritional status prior to clipping (Gruntman et al., 2011). A hypothesis to explain the lack of effect of the nitrogen treatment, is that the floral bud damage were not drastic enough to induce the growth of primary axes and thus to observe contrasted behavior between low and high nitrogen fertilization.

We identified common trends in the changes of branching and biomass allocation over the VN combinations. The VN showed differences in the magnitude of the responses that are difficult to interpret because of the inter-plant variability. However, VN responses seemed to vary according to clipping treatments. Both inflorescence and bud clippings were performed to simulate the reproductive damage that are commonly observed in WOSR crops. Frost event can lead to apical meristems losses in small plants. Cabbage stem flea beetles can also damage the apical meristems when feeding in the main stem in some occasions. However, inflorescence losses are less frequent than bud losses caused by the numerous flights of pollen beetles on WOSR crops. When only the flower buds were removed (ClipFB), Pollen and Gamin were more reactive in terms of branching than Exocet. When the whole inflorescences were removed (ClipInflo), VN responses were different with Exocet being the most reactive genotype. However, we failed to identify statistically significant relationship between the intensity of the responses in branching, biomass allocation, and the plant compensation. It is difficult to link directly a number of branches to tolerance. Plasticity may better be assessed through the rate of response or the precocity in the response. This needs dynamic characterization of the morphogenesis with non-destructive measurements on plants (e.g., dynamics of the number of branches or numbers of pods; Pinet, 2010).

Result obtained with the allometry relationships between the pods dry mass and number of pods illustrates the kind of trait we could define and provide for breeding programs. Our results indicate that there is a slow-down in the increase in the number of pods above a certain dry mass allocated to the pods for the primary inflorescences. Above this threshold, supplemental biomass will be affected to pod weight and not to the production of new pods. This threshold in pod dry mass has been observed for the primary inflorescences of ClipInflo but not for the secondary inflorescences under ClipInflo or with ClipFB. This shift in the biomass allocation could be explained by the competition between growing pods and the meristem that produces new pods. In secondary inflorescences, the number of pods increases linearly with pod dry mass indicating no competition between the production of new pods and the filling of existing pods. An increase in the slope of this relation has been detected in 13 combinations of VN and clipping treatments (among the 17 combinations of VN and clipping treatments with significant variation in the slope and/or the intercept). This increase could be linked to a capacity to produce a larger number of pods per unit of dry mass in response to clipping and might also correspond to a transient response by the plant to cutting and biomass reallocation. Since results evidenced that number of pods is the main lever for compensation (Figure 2), the number of pods produced per gram of pod dry mass could thus be an interesting trait to target for the selection of tolerant genotypes.

As regards with biomass allocation within the plant, the global trend upward in the shoot/root ratio in response to clipping treatments, while not significant, was suggestive of biomass reallocation from root compartment to aerial parts of the plant. This assumption is consistent with the functional equilibrium hypothesis: the dry matter distribution between root and shoot is regulated by equilibrium between root activity and shoot activity (Brouwer and De Wit, 1969). Thus, the production of new inflorescences and pods may have been supported by the biomass remobilization from roots to shoots. Change in biomass allocation from roots to shoots was also identified as a trait implied in the compensation of floral damage of annual wild species (common groundsel—Obeso and Grubb, 1994; coast tarweed—Gonzales et al., 2008; Carolina horsenettle—Wise and Cummins, 2006). However, other plant compartments could contribute to biomass supply to the inflorescences and the pods. In our study, data did not enable a detailed exploration of biomass reallocation from the stems and leaves based on variations in leaf mass per area that are highly variable and decrease during plant bolting and branching (Jullien et al., 2009). Similarly, it was not possible to evaluate adaptations regarding the number of seeds per pod, even though these have been shown to change in line with the source/sink ratio (Wang et al., 2011).

The harvest index is an integrated final criterion to assess the balance of the biomass allocation between pods and the rest of the plant (except the root compartment). In our study it was either maintained (for inflorescence cutting) or decreased (for floral bud clipping). The decrease in harvest index can be related to the cost in biomass for the production of new axes and inflorescences that bear the new pods. In the case of inflorescence clipping, the harvest index was not affected because initial flower stalk was also clipped and did not grow out avoiding assimilates investment in a non-productive axis. The new axes are replacement after a kind of reset of the axis. On the contrary, in the case of floral bud clipping, only the buds have been cut and initial flower stalk has been maintained. The compensative inflorescences are in addition. The cost of the production of axes on the global sources/sink ratio of the plant has been evaluated by a modeling approach by Jullien et al. (2012) and Pinet (2010). Simulations show that the plant bolting and the elongation of the ramification induce a dramatic decrease in the sources/sink ratio largely prior seed filling. In the case of flower damage, simulations show that the sources/sink ratio increases temporarily because of the loss of sink organs. However, a quick return to normal value of the sources/sink ratio is observed following the growth of new sink organs (axes and pods).

Our study explores the interactions between morphogenesis and biomass distribution shown in the conceptual diagram of Figure 1 in the case of flower damage in WOSR. A next step could be to focus on the change in the distribution of biomass after clipping and the ability of the plant to reuse this biomass to produce new pods. This may provide new traits to be target in breeding programs in order to improve WOSR tolerance to floral bud damage.

### Conflict of interest statement

The authors declare that the research was conducted in the absence of any commercial or financial relationships that could be construed as a potential conflict of interest. The Reviewer Sabine Demotes-Mainard declares that, despite being affiliated to the same institution as author Amélie Pinet, the review process was handled objectively and no conflict of interest exists.
