# Supplementary material for: Floral bud damage compensation by branching and biomass allocation in genotypes of Brassica napus with different architecture and branching potential
Source: Front Plant Sci. 2015 Feb 24;6:70. doi: 10.3389/fpls.2015.00070 (PMC4338677; doi:10.3389/fpls.2015.00070)
Supplement: Supplementary file 1 [file DataSheet1.PDF]

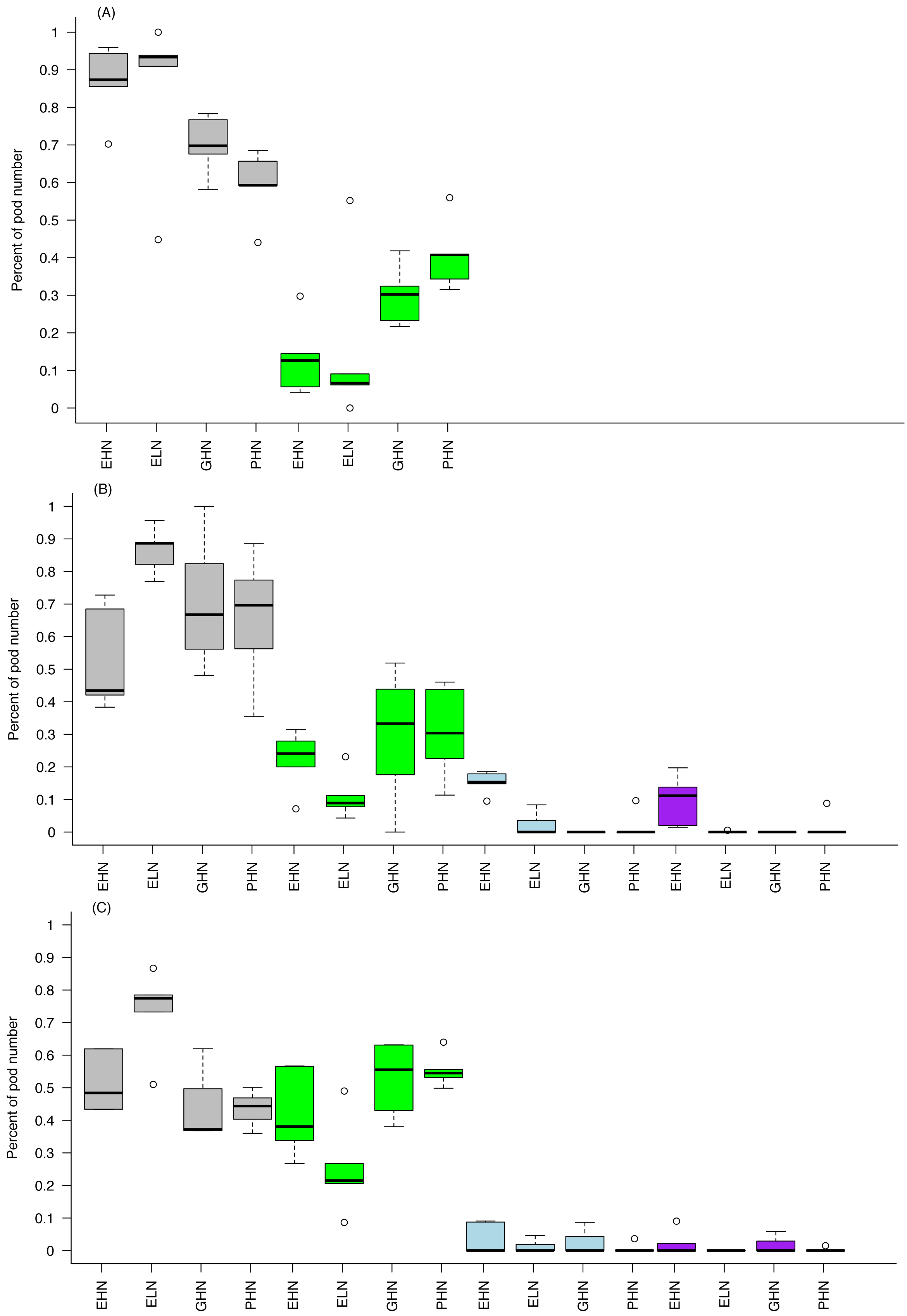

Figure 1 SI. Pods distribution between different axes of the plant for ClipInflo. Control (A), ClipI0 (B) and ClipI4 (C). Grey boxplots correspond to pods of primary inflorescences carried by existing primary axes. Green boxplots correspond to pods of secondary inflorescences

carried by existing primary axes. Blue boxplots correspond to pods of primary inflorescences carried by new primary axes. Purple boxplots correspond to pods of secondary inflorescences carried by new primary axes. Results are expressed as a percentage of the total number of pods.

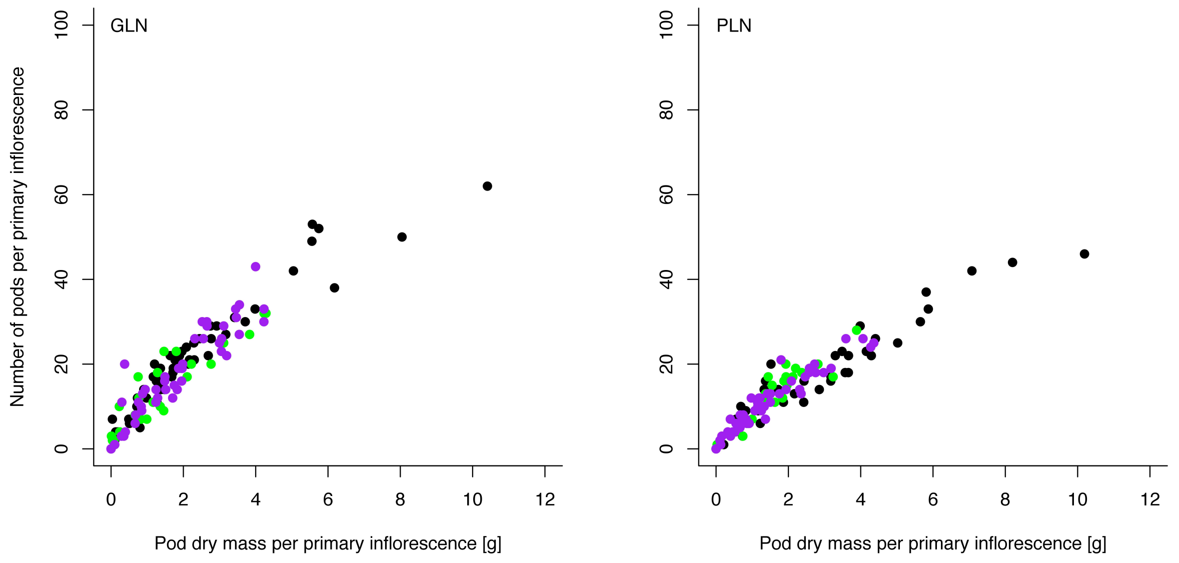

Figure 2 SI. Relationship between the number of pods and the pod dry mass on the primary inflorescences for GLN and PLN of ClipInflo. Black, green and purple dots correspond to Control, ClipFB4 and ClipFB7 treatments, respectively.

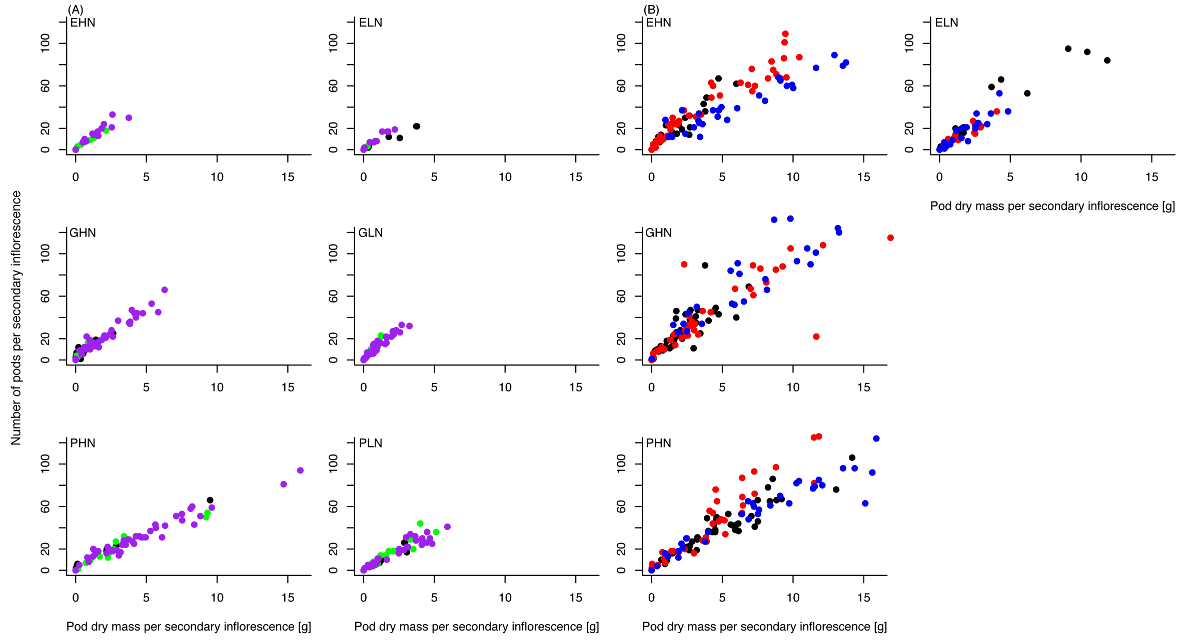

Figure 3 SI. Relationship between the number of pods and the pod dry mass on the secondary inflorescences for EHN, ELN, GHN, GLN, PHN, PLN of ClipFB (A) and for EHN, ELN, GHN and PHN of ClipInflo (B). Black, green and purple dots correspond to Control, ClipFB4

and ClipFB7 treatments, respectively. Black, red and blue dots correspond to Control, ClipI0 and ClipI4 treatments, respectively.
